# SMARCE1 regulates metastatic potential of breast cancer cells through the HIF1A/PTK2 pathway

**DOI:** 10.1186/s13058-016-0738-9

**Published:** 2016-08-05

**Authors:** Aarti Sethuraman, Martin Brown, Tiffany N. Seagroves, Zhao-Hui Wu, Lawrence M. Pfeffer, Meiyun Fan

**Affiliations:** 1 Department of Pathology and Laboratory Medicine and Center for Cancer Research, University of Tennessee Health Science Center, Memphis, TN 38163 USA; 2 Department of Pathology and Laboratory Medicine and Center for Cancer Research, 19 South Manassas Street, Memphis, TN 38163 USA

**Keywords:** SMARCE1/BAF57, HIF1A, PTK2/FAK, Anoikis, Breast cancer

## Abstract

**Background:**

While aberrant activation of the chromatin-remodeling SWI/SNF complexes has been associated with cancer development and progression, the role of each subunit in tumor cells is poorly defined. This study is aimed to characterize the role of SMARCE1/BAF57 in regulating metastasis of breast cancer cells.

**Methods:**

Genetic approaches and chemical inhibitors were used to manipulate the activities of SMARCE1 and its downstream targets in multiple breast cancer cell lines. Xenograft mouse models were used to analyze the role of SMARCE1 in lung metastasis in vivo. Nonadherent culture conditions were used to elucidate the role of SMARCE1 in regulating anoikis. Chromatin immunoprecipitation (ChIP), immunoprecipitation, and immunoblotting assays were designed to dissect the mechanism of action of SMARCE1. Public databases were used to investigate the relationship between SMARCE1 deregulation and breast cancer prognosis.

**Results:**

SMARCE1 knockdown reduced lung metastasis of breast cancer cells and sensitized tumor cells to anoikis. In response to loss of attachment, SMARCE1 interacted with and potentiated transcriptional activity of HIF1A, resulting in rapid PTK2 activation. Both HIF1A and PTK2 were indispensable for SMARCE1-mediated protection against anoikis by promoting activation of ERK and AKT pathways while suppressing the expression of pro-apoptotic BIM protein. Expression data analysis of a large cohort of human breast tumors revealed that high expression of SMARCE1 or PTK2 is associated with poor prognosis and tumor relapse, and PTK2 expression is positively correlated with SMARCE1 expression in basal-like and luminal B subtypes of breast tumors.

**Conclusions:**

SMARCE1 plays an essential role in breast cancer metastasis by protecting cells against anoikis through the HIF1A/PTK2 pathway. SMARCE1-mediated PTK2 activation likely plays a key role in promoting metastasis of basal-like and luminal B subtype of breast tumors.

## Background

Breast cancer is the most frequently diagnosed form of cancer in American women and is the second leading cause of cancer-related deaths, primarily due to the incurable nature of metastatic breast cancer [1]. Metastasis is the process whereby cancer cells spread from the site of the original tumor to distant organs [2]. A stepwise sequence of events is involved in the metastatic process, whereby tumor cells encounter a number of environmental challenges such as loss of attachment to the extracellular matrix, physical stress during circulation in blood/lymph vessels, and immune surveillance [3, 4]. Only a small fraction of disseminated cells are able to alter gene expression patterns to obtain new phenotypic features according to environmental cues, and ultimately survive and adapt to form metastatic lesions.

Chromatin remodeling plays a key role in tumor progression by altering gene expression patterns to facilitate rapid adaptation of tumor cells to extracellular stimuli [5]. Aberrant chromatin remodeling activity is frequently detected in human tumors. High-throughput genome and/or exome sequencing studies revealed that the SWI/SNF (switching-defective and sucrose nonfermenting) chromatin-remodeling complexes are the most highly mutated chromatin regulatory complexes, with nearly 20 % of human cancers harboring mutations in at least one of the genes encoding SWI/SNF subunits [6]. Human SWI/SNF complexes consist of either Brahma-related gene 1 (BRG1)/SMARCA4 or Brahma gene (BRM)/SMARCA2 subunit that possesses helicase and ATPase activity, and several BRG/BRM associated factors (BAFs) that regulate the DNA-binding specificity and affinity of the complexes [6, 7]. The cancer-associated mutations cause aberrant activation of the residual SWI/SNF complexes due to distinct subunit configurations, rather than eliminating the chromatin-remodeling activity [8–10]. Although earlier studies identified several SWI/SNF subunits (e.g*.*, SMARCB1 and SMARCA4) as tumor repressors, emerging evidence suggests that SWI/SNF activity is essential for tumor initiation, maintenance and progression [10, 11]. Therefore, it becomes important to define the role of each SWI/SNF subunit in development and progression of various types of tumors.

Genomic amplification of BAF57/SMARCE1, a core subunit of human SWI/SNF complexes that plays a role in interaction with transcription factors and chromatin, has been linked to high risk of recurrence of estrogen/progesterone receptor-negative breast tumors [12]. However, the underlying molecular mechanisms remain undefined. In this study, we demonstrated that SMARCE1 plays a key role in breast cancer metastasis by protecting breast cancer cells against anoikis through the HIF1A/PTK2 pathway.

## Methods

### Cell culture

All cell lines were purchased from ATCC (Manassas, VA, USA). MDA-MB-231, HCC38, BT549 and their derivatives were maintained in Minimum Essential Media (Thermo Fisher Scientific, Rockford, IL, USA) supplemented with 10 % fetal bovine serum (FBS), 200 units/ml penicillin-streptomycin (Cellgro, Manassas, VA, USA) and 0.5 μg/ml amphotericin B (Cellgro, Manassas, VA, USA). 184B5 cells were cultured in Mammary Epithelial Cell Growth Medium (MEGM, Lonza, Basel, Switzerland). A lung metastatic derivative of MDA-MB-231 (LM) was established as previously described [13]. Cells stably expressing short hairpin RNA (shRNA) against SMARCE1, HIF1A, PTK2 or empty vector (EV) control were established by lentivirus transduction and selected in medium containing 2 μg/ml puromycin (Sigma-Aldrich, St. Louis, MO, USA). pLKO.1-HIF1AshRNA (NM_001530.x-1048s1c1), pLKO.1-SMARCE1shRNA (TRCN0000015779) and pLKO.1-PTK2shRNA (TRCN0000344599) were obtained from Open Biosystems/GE Dharmacon (Lafayette, CO, USA). Cells stably expressing focal adhesion kinase (FAK)/PTK2 or SMARCE1 were obtained by lentiviral transduction and selected in medium containing 30 μg/ml blasticidine (Sigma-Aldrich). pLX304-PTK2 (HsCD00442503) and pLX304-SMARCE1 (HsCD00440078) were purchased from DNASU Plasmid Repository (Tempe, AZ, USA). Control cells were established by lentivirus transduction to express selection marker only. Accell SMARTpool (a mixture of four short inhibitory RNA (siRNA) provided as a single reagent) for human SMARCE1 was obtained from Dharmacon (GE Healthcare Life Sciences, Pittsburgh, PA, USA) and used to knock down SMARCE1 expression by following the manufactory’s protocol. The Accell Human Control siRNA (non-targeting) was used as control oligonucleotides.

### Orthotopic xenograft model and experimental lung metastasis model (tail vein injection)

All animal studies adhered to protocols approved by the Institutional Animal Care and Use Committee of University of Tennessee Health Science Center. For the orthotopic xenograft model in NOD.Cg Prkdcscid Il2rgtm1Wjl/SzJ (NSG) mice (The Jackson Laboratory, Bar Harbor, ME, USA), cells (7.5 × 10^5^ cells in 10 μl phosphate-buffered saline) were surgically inoculated into the right inguinal mammary gland fat pads of 4-week-old female mice as previously described [14]. Mice were inspected weekly for tumor appearance by visual observation and palpation. Primary tumor outgrowth was monitored twice a week using digital calipers. Tumor volume was calculated as: volume = (width^2^ × length)/2. Tumor and lung tissues were extracted 6 weeks after inoculation. The left lung lobes were imaged under the fluorescent microscope, fixed with 4 % paraformaldehyde, followed by tissue section (10 μm) and hematoxylin and eosin staining. Metastatic loci in lungs were quantified using ImageJ software. For experimental lung metastasis model, cells were inoculated into to 4-week-old NSG mice by tail vein injection. Whole blood was collected by cardiac puncture at various time and subjected to Ficoll-Paque separation (Ficoll-Paque PLUS, GE Healthcare Life Sciences, Piscataway, NJ, USA) to isolate circulating tumor cells. Fluorescent circulating tumor cells were counted under fluorescent microscope using a ×10 objective.

### Quantitation of mRNA using qPCR

Total RNA was prepared with Trizol (Life Technologies, Grand Island, NY, USA). cDNAs for mRNA were synthesized by using iScript cDNA Synthesis Kits (Bio-Rad, Hercules, CA, USA). qPCR was performed on the CFX96^™^ Real-Time PCR Detection System using SYBR Green Supermix (Bio-Rad). Expression data of mRNA were normalized by the 2-ΔΔCT method to RPL13A, and presented as mean ± SE (*n* = 3). qPCR primers were obtained from PrimerBank [15].

### Migration and invasion assays

Cells (20,000 cells/0.5 ml/well) were plated onto control membrane inserts with 8-micron pores or Matrigel-coated membrane transwell inserts (BD Biosciences, Bedford, MA, USA), which are placed in 24-well chambers filled with 0.6 ml growth medium. Twenty-four hours after plating, cells that remained on the upper surface of the membrane were removed by cotton-tipped swabs, and cells that migrated/invaded to the lower surface of the membrane were viewed under the fluorescent microscope. The percent migration was expressed as percent migration = (mean number of cells migrating through Matrigel insert membrane × 100/mean seeding control) and percent invasion was expressed as: percent invasion = (mean number of cells invading through Matrigel insert membrane × 100/mean number of cells migrating through control insert membrane.

### Suspension culture and inhibitor assays

To mimic loss of attachment, cells were seeded in culture dishes coated with polyHEMA (Sigma-Aldrich) to prevent cell adherence for various period of time. The cell suspensions were placed into regular culture dishes 16 h prior to end-point viable cell counting. To identify the signaling pathways that promote survival of detached cells, a panel of chemical inhibitors were added in culture medium: PTK2/FAK inhibitor – 0.5 μM PF562271 (Selleckchem, Houston, TX, USA), ERK1/2 inhibitor – 10 μM U0126 (LC Laboratories, Woburn, MA, USA) or 10 μM PD98059 (Santa Cruz Biotechnology, Dallas, TX, USA), AKT inhibitor – 30 μM ZSTK474 (LC Laboratories), TGFBRI/II inhibitor – 10 μM LY210761 (Selleckchem), SRC inhibitor – 3 μM dasanitib (LC Laboratories) or Notch inhibitor – 5 μM DAPT (Selleckchem).

### Protein extraction and immunoblotting

Protein extraction from whole cell lysates, cytosolic and nuclear fractions, and immunoblotting with luminescence detection were performed as previously described [13]. The following antibodies were used in this study: anti-PTK2/FAK, FAK-Tyr576P/Tyr577P, ERK1/2-Thr202P/Tyr204P, ERK1/2, AKT-Tyr416P, AKT, and BCL2L11 from Cell Signaling Technologies (Boston, MA, USA); anti-HIF1A from Novus Biologicals (Littleton, CO, USA); anti-SMARCE1 and SMARCA4 from Bethyl Laboratories (Montgomery, TX, USA); anti-GAPDH from Millipore (Merck, Darmstadt, Germany) and anti-TBP from Abcam (Cambridge, MA, USA).

### Chromatin immunoprecipitation (ChIP)

Cells (2 × 10^7^) were crosslinked with 1 % formaldehyde (Pierce^™^ 16 % formaldehyde (w/v), methanol-free, Life Technologies) for 15 minutes. The nuclear fraction was prepared using the NE-PER^™^ Nuclear and Cytoplasmic Extraction Reagents (Life Technologies), chromatin was sheared by sonication to obtain approximately 500 bp fragments and the soluble fraction was collected after centrifuge (12,000 rpm, 15 min). The soluble nuclear fractions were pre-cleaned with control IgG and MagnaBind Goat anti-Rabbit IgG (Life Technologies) and used for immunoprecipitation with antibodies against HIF1A, SMARCE1 or SMARCA4. Immunocomplexes were isolated with MagnaBind Goat anti-Rabbit IgG, de-crosslinked, and subjected to DNA preparation using the MiniElute PCR Purification Kit (QIAGEN, Germantown, MD, USA). qPCR was performed to detect the presence of PTK2 promoter region by using primers (Forward: 5′-CTCTTCCTCCTCCTGCCTCT-3′; reverse 5′-GTTCGGGGAAGACAGAAA GG-3′). To detect protein-protein interaction, the immunocomplexes were dissolved in Laemmli Sample Buffer (Bio-Rad) and subjected to immunoblotting assay.

### Caspase assay

A total of 5 × 10^5^ cells were plated in polyHEMA-coated dishes and caspase-3 levels were measured at indicated time points by using the Caspase-Glo® 3/7 assay kit (Promega, Madison, WI, USA). Caspase activity was calculated as per manufacturer protocol, normalized to cell numbers.

### Nucleosome scanning assay (NUSA)

10^6^ cells were placed under suspension culture in polyHEMA-coated dishes for 0, 0.5, 1 and 2 hours. Nuclei were isolated, treated with Atlantis double-strand specific DNase (dsDNase) and subjected to DNA purification according to the EZ Nucleosomal DNA Prep Kit (Zymo Research, Irvine, CA, USA). qPCR was performed to determine nucleosome positioning on the *PTK2* promoter. Overlapping primers were designed from −150 to +1589 relative to start site of *PTK2* promoter to generate amplicons of approximately 150 bp, the size of DNA covered by one nucleosome. DNA amount was calculated according to a standard curve (qPCR CTs vs*.* various concentrations of template) generated for each primer and normalized to qPCR CTs of DNA purified from equal number of nuclei untreated with dsDNase.

### Statistical analysis

Analysis of variance (ANOVA) and post hoc least significant difference analysis or *t* tests were performed using GraphPad Prism 5 software (Graphpad, San Diego, CA, USA). *p* values < 0.05 (*) were considered statistically significant. Data from two or three independent experiments with replicates are presented as means ± standard deviation (SD).

## Results

### SMARCE1 knockdown reduces lung metastasis of breast cancer in vivo

**Fig. 1:**
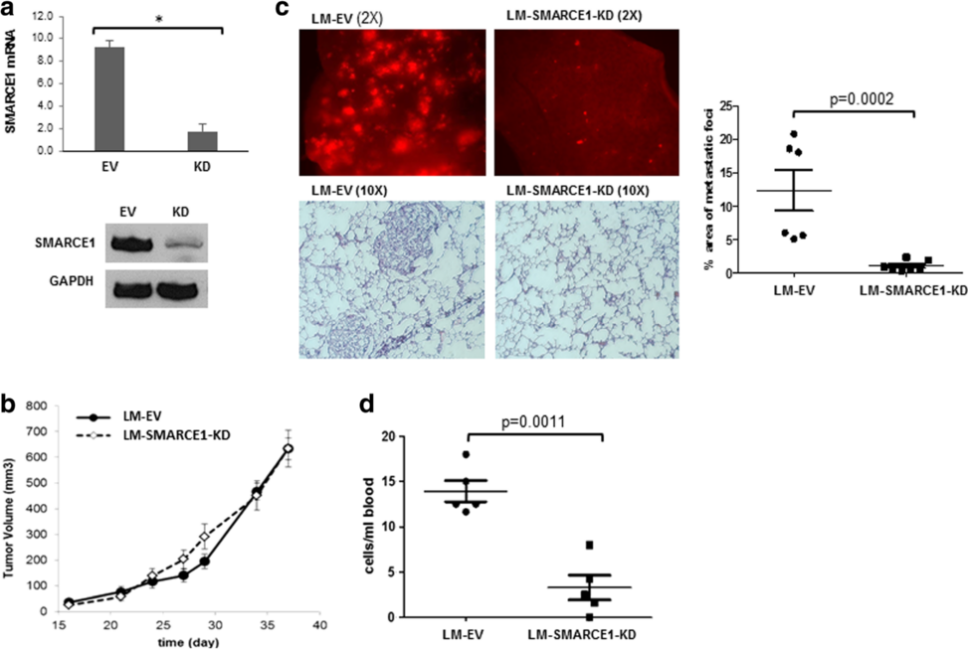
SMARCE1 knockdown reduces lung metastasis of breast cancer in vivo. **a** Expression levels of SMARCE1 mRNA and protein in LM-EV and LM-SMARCE1-KD cells. **b** Effect of SMARCE1 knockdown on the growth of xenografts in the fourth inguinal mammary fat pads of female NSG mice. **c** Spontaneous lung metastasis from orthotopic sites. Metastatic foci of tumor cells expressing red fluorescent protein on the dorsal surface of the left lung lobe were imaged 38 days after tumor cell inoculation (*upper panel*). The presence of tumor cells in the lungs was visualized by hematoxylin and eosin (H&E) staining of formalin-fixed lung sections (10 μM) and quantified by ImageJ software (*lower panel*). **d** Number of circulating tumor cells in blood. Fluorescent tumor cells in mouse blood were isolated and counted 38 days after tumor cell inoculation. *EV* empty vector, *KD* knockdown, *LM* lung metastatic cell line derived from MDA-MB-231

To define the role of SMARCE1 in breast cancer metastasis, we examined the effect of SMARCE1 knockdown (KD) on spontaneous lung metastasis using an orthotopic xenograft mouse model derived from a lung metastatic variant of MDA-MB-231 cells, which was previously described and designated as LM [13]. SMARCE1 knockdown showed no significant effect on the latency and growth rate of primary xenografts in mammary gland fat pads (Fig. 1a and b, LM-SMARCE1-KD vs*.* LM-EV), but substantially reduced both the number and size of metastatic foci in lungs (Fig. 1c, LM-SMARCE1-KD vs*.* LM-EV). According to the images of lung tissue sections, metastatic foci occupied 12.30 ± 3.87 % of the lung parenchyma in mice 6 weeks after inoculation with LM-EV cells, which was reduced to 1.02 ± 0.76 % in mice inoculated with LM-SMARCE1-KD cells (*p* = 0.0002) (Fig. 1c bar graph). By examining the number of tumor cells in blood, we found that SMARCE1 knockdown significantly reduced the number of circulating tumor cells (*p* = 0.0011) (Fig. 1d). Together, these results suggest that SMARCE1 activity is dispensable for primary tumor outgrowth, but essential for distant metastasis of MDA-MB-231 cells.

### SMARCE1 knockdown reduces lung colonization of tumor cells inoculated through tail vein

**Fig. 2:**
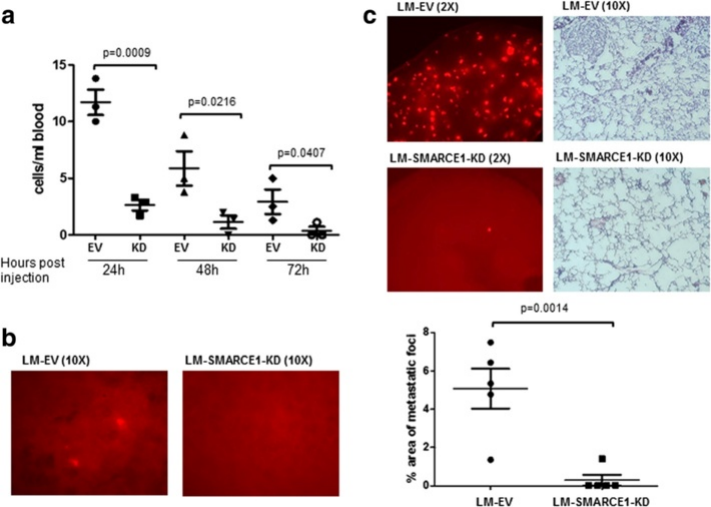
SMARCE1 knockdown reduces lung colonization of tumor cells inoculated through tail veins. **a** Number of circulating tumor cells in blood collected at various times after tail vein injection in NSG mice. **b** Fluorescent tumor cells in lungs of NSG mice 72 h after tail vein injection. Representative images of five lungs for each group were shown. **c** Fluorescent tumor foci in the left lung lobes of NSG mice 4 weeks after tail vein injection of tumor cells. The area of tumor foci on the dorsal surface of the left lung lobe was quantified by ImageJ

Metastasis is a multistep process involving local invasion, circulation, extravasation, colonization, and outgrowth of metastatic foci [16]. To identify the steps of the metastatic cascade that requires SMARCE1 activity, we examined the effect of SMARCE1 knockdown on the ability of tumor cells to survive circulation and colonize lungs by using an experimental metastasis model. LM-EV and LM-SMARCE1-KD cells (5 × 10^5^) were injected into the left lateral tail vein of 5-week-old female NSG mice. Tumor cells in the bloodstream and lung tissues were examined at various times after injection (Fig. 2a). As expected, the number of circulating tumor cells in blood decreased over time. Interestingly, at any given time point, the number of LM-EV cells in the bloodstream was significantly higher than that of the LM-SMARCE1-KD cells (Fig. 2a). At 72 hours past tail vein injection, we observed tumor cells in the lungs of mice inoculated with LM-EV cells but not in mice with LM-SMARCE1-KD cells (Fig. 2b). Four weeks post injection, a lower number of tumor foci was observed in lungs of mice inoculated with LM-SMARCE1-KD cells than that in mice with LM-EV cells (Fig. 2c). Together, these results suggest that SMARCE1 knockdown diminish the ability of tumor cells to survive circulation.

### SMARCE1 knockdown sensitizes tumor cells to anoikis

**Fig. 3:**
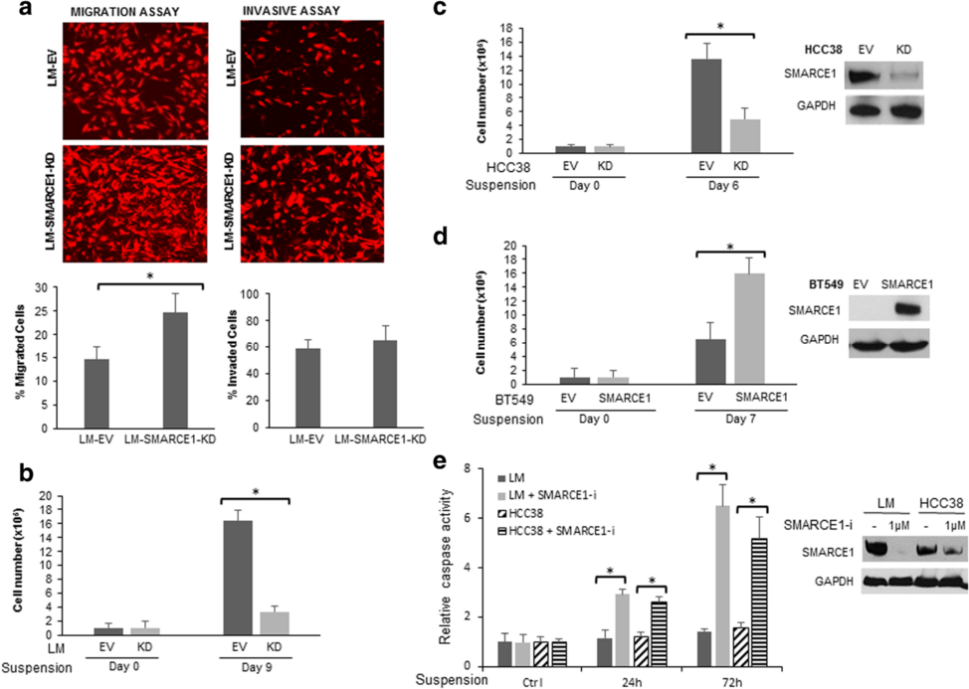
SMARCE1 knockdown promotes cell migration, but sensitize cells to anoikis. **a** Effect of SMARCE1 knockdown in LM cells on cell migration and invasion. Boyden chambers, uncoated or coated with Matrigel were used to measure cell migratory and invasive potential, respectively. **b** Effect of SMARCE1 knockdown in LM cells on viability of cells cultured in dishes coated with polyHEMA to prevent adhesion. **c** Effect of SMARCE1 knockdown in HCC38 cells. **d** SMARCE1 overexpression in BT549 cells. **e** Blocking SMARCE1 expression by siRNA enhanced detachment-induced caspase activation in LM and HCC38 cells. Accell SMARTpool of SMARCE1-siRNA (1 μM) were delivered into cells 48 h prior to detachment. Caspase activities were determined at indicated time points after cell detachment by using the Caspase-Glo 3/7 assay kit. *p < 0.05, Student *t* test. *EV* empty vector, *KD* knockdown, *LM* lung metastatic cell line derived from MDA-MB-231

Our in vivo experiment results implicate that SMARCE1 plays an essential role in distant metastasis of breast cancer cells by promoting the survival of circulating tumor cells. However, it is unclear whether SMARCE1 plays a role in early metastatic events such as cell migration and invasion. To examine the effect of SMARCE1 knockdown on the migratory and invasive potential of tumor cells, we performed Boyden chamber transwell assays. Cells were plated in the uncoated Boyden chamber to measure their migratory potential, then in a Matrigel-coated chamber to determine their invasiveness. As showed in Fig. 3a, SMARCE1 knockdown in LM cells significantly increased cell migratory potential, but showed no significant effect on cell invasiveness. This observation suggests that SMARCE1 activity is dispensable for cell migration and invasion. Next, we examined the effect of SMARCE1 knockdown on cell sensitivity to anoikis by monitoring cell viability after culture in dishes coated with polyHEMA to prevent attachment. As showed in Fig. 3b, SMARCE1 knockdown in LM cells substantially reduced the number of viable cells cultured in suspension. This result is consistent with our results from in vivo experiments and confirms that SMARCE1 plays a key role in protecting LM cells against anoikis.

To determine whether SMARCE1-mediated anoikis resistance can be extended to other breast cancer cell lines, we examine the effect of SMARCE1 knockdown in HCC38 (Fig. 3c), a triple-negative breast cancer cell line. We found that SMARCE1 knockdown resulted in significant reduction of the viability of cells in suspension culture. In addition, we examined the effect of SMARCE1 overexpression in BT549, a triple-negative breast cancer cell line that lacks SMARCE1 expression due to a biallelic inactivating mutation that causes a frameshift [17]. As expected, no SMARCE1 mRNA or protein was detected in BT549 cells (Fig. 3d). Enforced SMARCE1 expression in BT549 substantially increased the viability of cells in suspension culture. To validate the observations with SMARCE1-shRNA, we examined the effect of SMARCE1 siRNA (Accell SMARTpool, GE Dharmacon) or control oligonucleotides on anoikis in LM and HCC38 cell lines. As showed in Fig. 3e, SMARCE1 siRNA (SMARCE1-i) treatment resulted in decreased expression of SMARCE1 protein and enhanced activation of caspase 3/7 in cells under suspension culture. These results demonstrate that SMARCE1 plays role in anoikis resistance of breast cancer cells.

### SMARCE1-mediated anoikis resistance requires activation of PTK2, ERK and AKT pathways

**Fig. 4:**
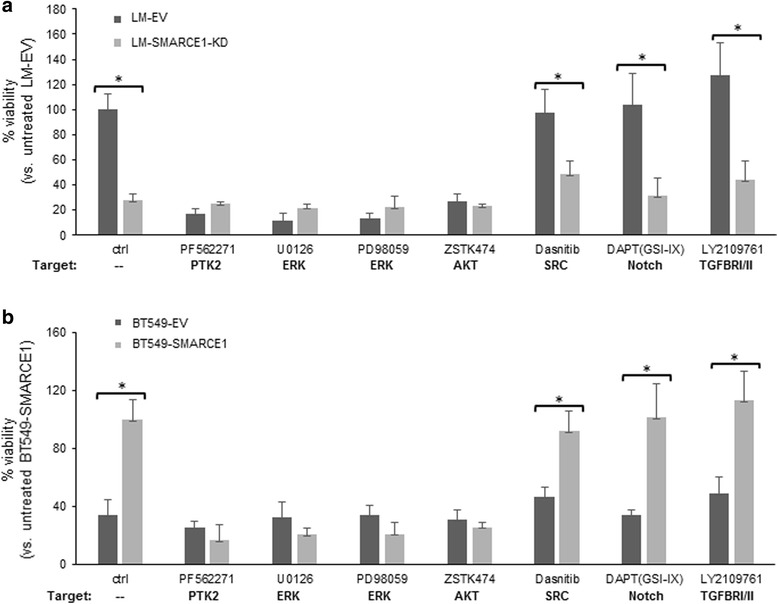
SMARCE1-mediated anoikis resistance requires activation of PTK2, ERK, and AKT pathways. **a** Viability of LM-EV and LM-SMARCE1-KD cells cultured in polyHEMA-coated dishes in the absence or presence of indicated inhibitors. **b** Viability of BT549-EV and BT549-SMARCE1 cells cultured in polyHEMA-coated dishes in the absence or presence of indicated inhibitors. For all experiments, viable cells were counted after 7 days in suspension culture. Concentration of inhibitors: 0.5 μM PF562271, 10 μM U0126, 10 μM PD98059, 30 μM ZSTK474, 3 μM dasanitib, 5 μM DAPT, and 10 μM LY2109761. *p < 0.05, Student *t* test. *EV* empty vector, *KD* knockdown, *LM* lung metastatic cell line derived from MDA-MB-231

**Fig. 5:**
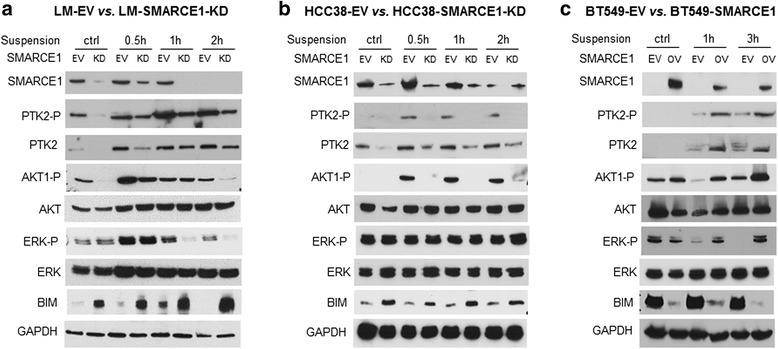
SMARCE1-dependent activation of PTK2, ERK, and AKT pathways by cell detachment. **a** Effect of SMARCE1 knockdown in LM cells on detachment-induced activation of pro-survival (FAK/PTK2, ERK, and AKT) and pro-apoptotic (BIM) signaling pathways. **b** Effect of SMARCE1 knockdown in HCC38 cells. **c** Effect of SMARCE1 overexpression in BT549 cells. The levels of protein expression and phosphorylation were determined by immunoblotting assays. GAPDH was included as a loading control. *EV* empty vector, *KD* knockdown, *LM* lung metastatic cell line derived from MDA-MB-231

To gain insights into the molecular mechanisms underlying SMARCE1-mediated anoikis resistance, we sought to identify signaling pathways regulated by SMARCE1 in detached cells by using chemical inhibitors of several signaling pathways that have been linked to anoikis resistance of cancer cells [18, 19]. As showed in Fig. 4a, inhibitors of focal adhesion kinase (PTK2/FAK), ERK, and AKT significantly reduced the viability of LM-EV cells in suspension culture. In contrast, these inhibitors showed no significant effect on LM-SMARCE1-KD cells. The same experiments were performed with BT549-EV and BT549-SMARCE1 cells and the result showed that SMARCE1-mediated increase of cell viability was effectively abolished by inhibitors of PTK2, ERK, and AKT (Fig. 4b). In agreement with the observed effects of chemical inhibitors on cell viability, immunoblotting analysis showed that LM-EV cells in suspension culture had higher levels of PTK2 protein, ERK phosphorylation, and AKT phosphorylation than LM-SMARCE1-KD cells (Fig. 5a). In addition, elevated expression of the pro-apoptotic protein BCL2-interacting mediator of cell death (BIM)/BCL2L11, a marker of onset of anoikis in breast cancer cells, was detected in suspension cultures of LM-SMARCE1-KD cells, but not in LM-EV cells (Fig. 5a). Similarly, higher levels of PTK2 protein and AKT phosphorylation, concomitant with lower levels of BIM protein, were observed in HCC38-EV cells as compared to HCC38-SMARCE1-KD cells in suspension culture (Fig. 5b). However, ERK phosphorylation was not affected by cell detachment or SMARCE1 knockdown in the HCC38-EV cells. In addition, elevated expression levels of PTK2 protein, ERK phosphorylation, and AKT phosphorylation, but diminished levels of BIM, were detected in BT549-SMARCE1 cells in comparison to control BT549-EV cells under suspension culture (Fig. 5c). Together, these results suggest that activation of PTK2, ERK, and AKT pathways is critical for breast cancer cells to survive anoikis, and SMARCE1 activity is required for the activation of these survival pathways in detached cells.

### SMARCE1 collaborates with HIF1A to activate PTK2 transcription in detached cells

**Fig. 6:**
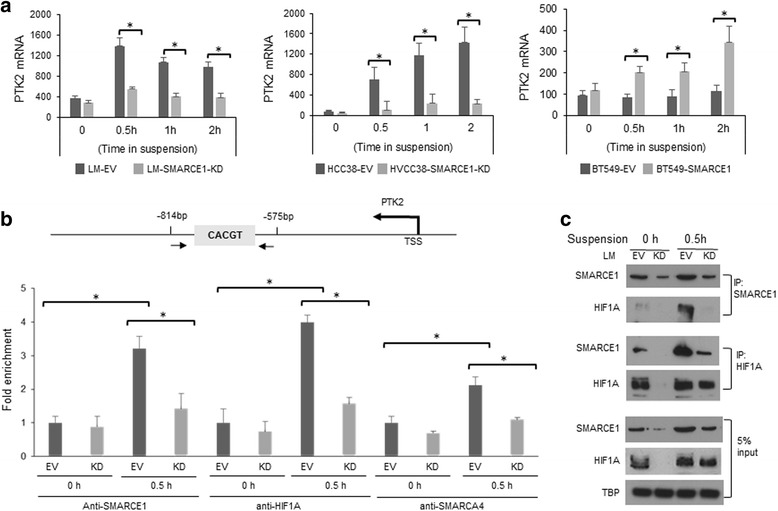
SMARCE1 collaborates with HIF1A to activate PTK2 transcription in detached cells. **a** Detachment-induced PTK2 mRNA expression is abolished by SMARCE1 knockdown in LM and HCC38 cells, whereas enhanced by SMARCE1 overexpression in BT549 cells. **b** Detachment-induced recruitment of HIF1A and SMARCA4 to the promoter region of PTK2 is diminished by SMARCE1 knockdown in LM cells. Chromatin binding of HIF1A, SMARCE1, and SMARCA4 was measured by ChIP-qPCR assay. Average fold enrichment of PTK2 promoter DNA by indicated antibodies (vs*.* control IgG) from three independent experiments were presented. **c** Detachment induces interaction between SMARCE1 and HIF1A in LM-EV and LM-SMARCE1-KD cells. The protein interaction was examined by immunoprecipitation/immunoblotting assays. *p < 0.05, Student *t* test. *EV* empty vector, *KD* knockdown, *LM* lung metastatic cell line derived from MDA-MB-231

Aberrant activation of PTK2, a non-receptor tyrosine kinase that is involved in focal adhesion, has been recognized as a key mediator of anoikis resistance in variety of tumor cells [18, 20]. For example, PTK2 activation was detected in circulating tumor cells isolated from 90 % of patients with metastatic breast tumors, implicating a role of PTK2 in breast cancer metastasis [21]. Therefore, we sought to examine whether SMARCE1 regulates PTK2 expression in detached cells. Based on immunoblotting and quantitative polymerase chain reaction (qPCR) analysis, we found that cell detachment induced PTK2 expression at both protein level (Fig. 5) and mRNA level (Fig. 6a) in a SMARCE1-dependent manner in LM, HCC38, and BT549 cells. This observation suggests that SMARCE1 is required for transcription activation of *PTK2* gene in detached cells.

By inspecting the transcription factor binding sequences in the proximal promoter region of *PTK2* gene (Fig. 6b), we found a potential HIF1A/ARTN binding site located in the binding region of SWI/SNF subunits (i.e*.*, SMARCA4 and SMARCB1) identified by ENCODE TFBS ChIP-seq analysis [22]. Normoxic HIF1A activation has been linked to anoikis resistance in breast cancer cells [21, 23]. In addition, SWI/SNF complexes were reported to play a role in modulating HIF1A-mediated transcription activation in cells under hypoxia [24, 25]. Therefore, we speculated that SMARCE1 regulates *PTK2* gene transcription through HIF1A in detached cells. By using ChIP-qPCR assay, we showed that cell detachment induced recruitment of SMARCE1, HIF1A, and BRG1/SMARCA4 to the *PTK2* promoter region (−874 to −575 bp) in LM-EV cells, which was abolished by SMARCE1 knockdown (Fig. 6b). In addition, reciprocal immunoprecipitation assays, we detected HIF1A and SMARCE1 proteins in the immunoprecipitated SMARCE1 and HIF1A complexes, respectively (Fig. 6c). These results suggest that SMARCE1 activity is required for HIF1A-mediated *PTK2* transcription activation in detached cells.

**Fig. 7:**
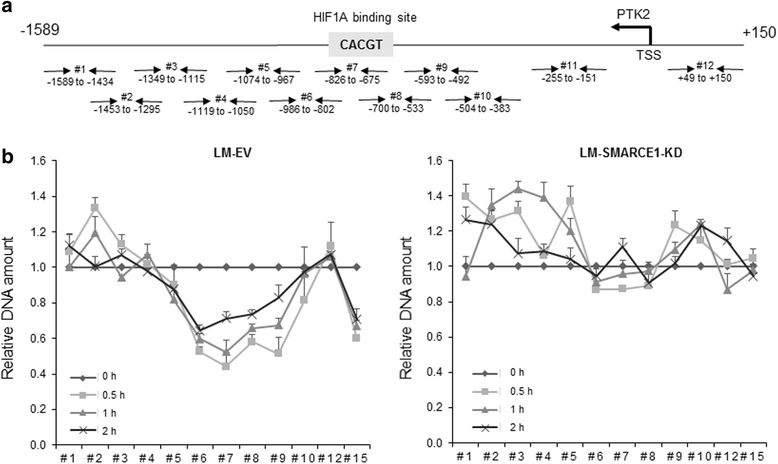
SMARCE1 orchestrates chromatin remodeling of PTK2 promoter in detached cells. **a** Overlapping primer designed for the nucleosome scanning assay and their amplicon sizes. The location of each primer set is given relative to the transcription start site (TSS). **b** Significant nucleosomal displacement is observed on PTK2 promoter (from −802 to −492 bp) in LM-EV cells after 0.5, 1, and 2 hours under suspension culture (vs. adherent cells), but not in LM-SMARCE1-KD cells. Results from three independent experiments were presented as means ± SE. *EV* empty vector, *KD* knockdown, *LM* lung metastatic cell line derived from MDA-MB-231

To examine the consequence of SMARCE1 and HIF1A binding to the *PTK2* promoter in detached cells, nucleosome scanning assay (NUSA) was performed to examine nucleosomal positioning on the *PTK2* promoter. We found that cell detachment induced nucleosomal disassembly on the *PTK2* promoter region bound by HIF1A and SMARCE1, indicated by decreased amount of DNA detected by qPCR as a result of increased sensitivity of *PTK2* promoter to dsDNase digestion (Fig. 7). However, cell detachment did not increase dsDNase sensitivity in LM-SMARCE1-KD cells (Fig. 7b, right panel). These observations provide evidence supporting a role of SMARCE1 in HIF1A-mediated *PTK2* transcriptional activation by chromatin remodeling in detached cells.

### HIF1A activates *PTK2* transcription and downstream activation of survival pathways in detached cells

**Fig. 8:**
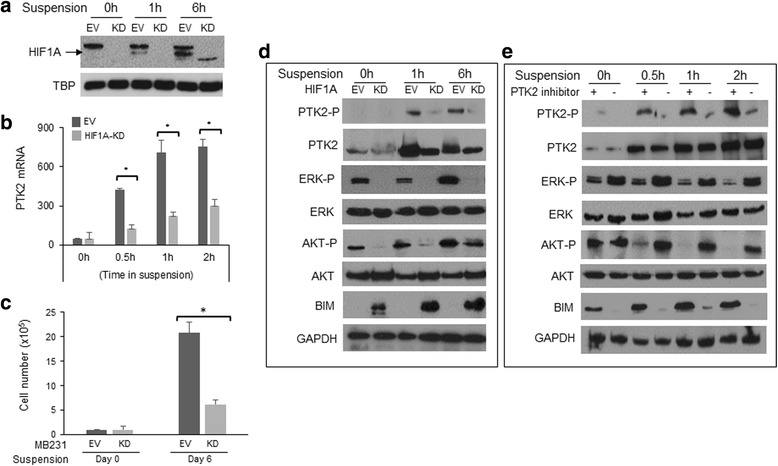
HIF1A activates PTK2 transcription and downstream survival pathways in detached cells. **a** Detachment induced HIF1A protein accumulation is eliminated by HIF1A knockdown in MDA-MB-231 cells. **b** HIF1A knockdown abolishes detachment induced PTK2 mRNA expression in MDA-MB-231 cells. **c** HIF1A knockdown sensitizes MDA-MB-231 cells to anoikis. Viable cells in suspension culture for 10 days were counted. **d** Effect of HIF1A knockdown in MDA-MB-231 cells on detachment-induced activation of pro-survival (PTK2, ERK, and AKT) and pro-apoptotic (BIM) signaling pathways. **e** Effect of PTK2 inhibition in MDA-MB-231 cells on detachment-induced activation of pro-survival and pro-apoptotic signaling pathways. The mRNA levels were measured by qPCR. The levels of protein expression and phosphorylation were determined by immunoblotting assays with GAPDH included as a loading control. *p < 0.05, Student *t* test. *EV* empty vector, *KD* knockdown, *LM* lung metastatic cell line derived from MDA-MB-231

To confirm that HIF1A-mediated *PTK2* transcription is critical for breast cancer cell to survive anoikis, we examined the effect of HIF1A knockdown on cell sensitivity to anoikis and detachment-induced activation of PTK2, ERK, and AKT. HIF1A knockdown in MDA-MB-231 cells effectively dampened detachment-induced HIF1A protein accumulation and *PTK2* mRNA expression (Fig. 8a and b), and reduced the number of viable cells in suspension culture (Fig. 8c). Immunoblot analysis showed that the levels of PTK2, phosphorylated-PTK2 (pTyr576/577), phosphorylated-ERK (pThr202/Tyr204), and phosphorylated-AKT (pTyr416) were significantly lower in HIF1A-KD cells than that in control MDA-MB-231-EV cells under suspension culture (Fig. 8d). Conversely, the pro-anoikis protein BIM was induced only in detached HIF1A-KD cells. To confirm that ERK and AKT phosphorylation are downstream events of PTK2 activation in detached cells, we examined the effect of PTK2 inhibition by PF562271 on activation of these pathways. As showed in Fig. 8e, pre-treatment with PF562271 effectively abolished detachment-induced phosphorylation of PTK2, ERK, and AKT. Taken together, these results suggest that HIF1A-mediated PTK2 transcription activation plays a key role in protecting cells against anoikis by sustaining the activation of survival signaling pathways (i.e., ERK and AKT), as well as suppressing pro-apoptotic signaling (i.e., BIM).

**Fig. 9:**
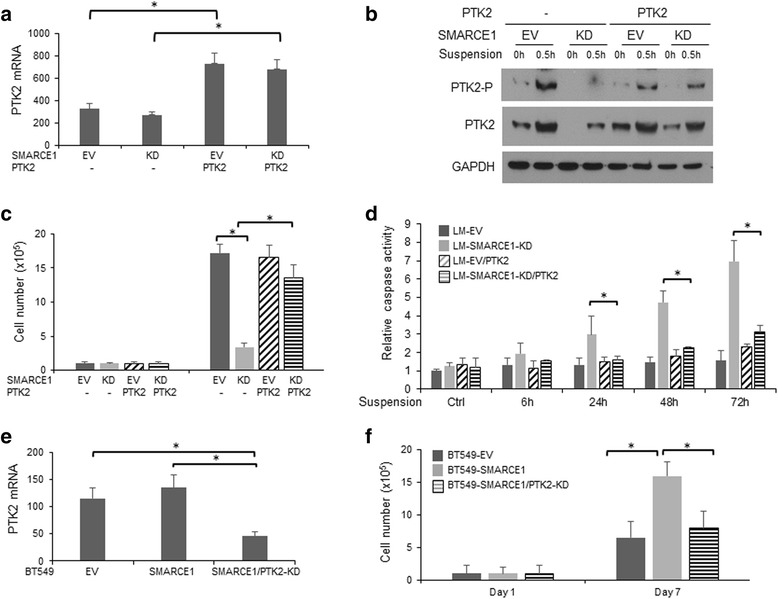
PTK2 activation is essential for SMARCE1-mediated anoikis resistance in breast cancer cells. **a** Expression levels of PTK2 mRNA in LM derivatives. **b** PTK2 protein expression and phosphorylation in LM derivatives cultured in polyHEMA-coated dishes. **c** Effect on PTK2 overexpression on viability of LM derivatives cultured in polyHEMA-coated dishes. **d** Effect on PTK2 overexpression on caspase activation in LM derivatives cultured in polyHEMA-coated dishes. Caspase activity was normalized to cell numbers and results from three independent experiments were presented as means ± SE. **e** Expression levels of PTK2 mRNA in BT549 derivative. **f** Effect of PTK2 knockdown on viability of BT549 derivative cultured in polyHEMA-coated dishes. *p < 0.05, Student *t* test. *EV* empty vector, *KD* knockdown, *LM* lung metastatic cell line derived from MDA-MB-231

To corroborate the role of PTK2 in SMARCE1-mediated anoikis resistance, we examined the effects of PTK2 overexpression and PTK2 knockdown on anoikis sensitivities of LM-SMARCE1-KD and BT549-SMARCE1 cells, respectively. We found that PTK2 overexpression effectively rescued LM-SMARCE1-KD cells from anoikis, indicated by increased number of viable cells and reduced caspase activation (Fig. 9a–d). Conversely, PTK2 knockdown abolished SMARCE1-mediated protection of BT549 cells against anoikis (Fig. 9e–f).

### SMARCE1 knockdown sensitizes non-tumorigenic mammary epithelial cells to anoikis

**Fig. 10:**
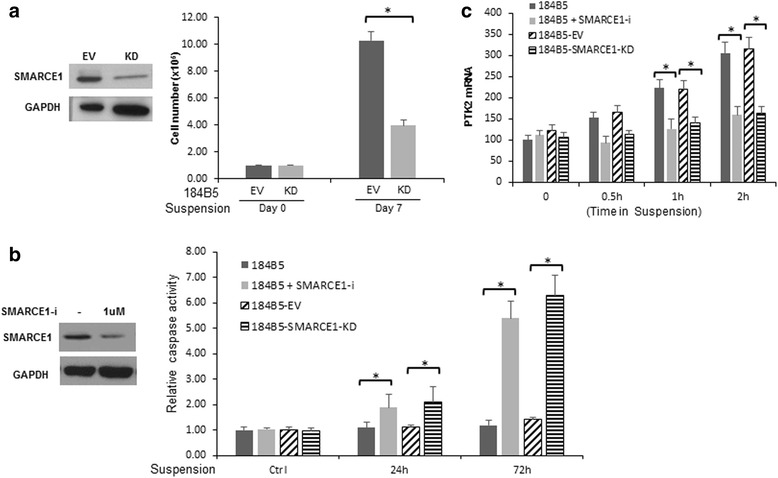
Blocking SMARCE1 expression sensitizes non-tumorigenic mammary epithelial cells to anoikis. **a** Effect of SMARCE1 knockdown by stable shRNA expression on viability of 184B5 cells cultured in polyHEMA-coated dishes. **b** Effect of stable shRNA expression (SMARCE1-KD) and SMARCE1-siRNA delivery (SMARCE1-i) on caspase activation in 184B5 cells cultured in polyHEMA-coated dishes. Caspase activity was measured by using the Caspase-Glo 3/7 assay kit and normalized to cell numbers. The results from three independent experiments were presented as means ± SE. **c** Detachment-induced *PTK2* mRNA expression is abolished by SMARCE1-KD or SMARCE1-i in 184B5 cells. mRNA levels were measured by qPCR. *EV* empty vector, *KD* knockdown

To examine whether the SMARCE1-dependent anoikis resistance occur at early stage of tumorigenesis, we examined the effect of SMARCE1 knockdown on anoikis sensitivity of 184B5, a mammary epithelial cell line immortalized by benzo(a)pyrene exposure [26]. Previous studies showed that 184B5 was not tumorigenic in immunosuppressed mice, but did form colonies in semisolid medium [26]. Therefore, 184B5 represents cells at early stage of tumorigenesis. We found that SMARCE1 inhibition in 184B5 cells, by either stable knockdown through shRNA expression (Fig. 10a) or transient siRNA delivery (Fig. 10b), blocked detachment-induced PTK2 upregulation (Fig. 10c) and sensitized cells to anoikis, indicated by reduced cell viability and increased caspase activation (Fig. 10a and b, respectively). These observations suggest that SMARCE1/PTK2-mediated anoikis resistance is an early event of tumorigenesis.

### Higher SMARCE1 and PTK2 expression is associated with poor prognosis of patients with basal-like or luminal B subtype of breast tumors

**Fig. 11:**
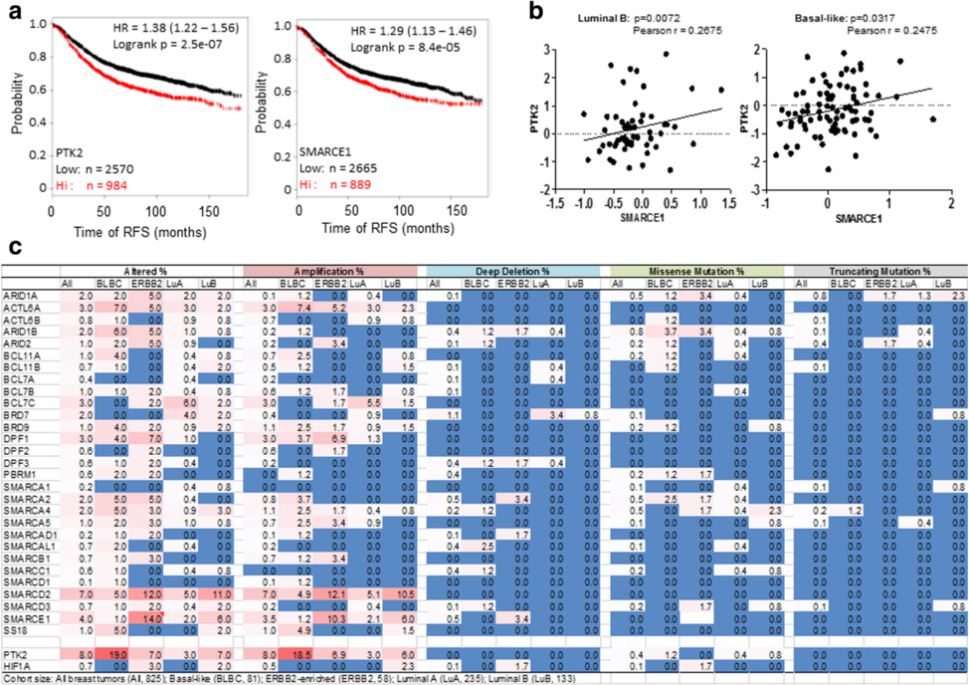
Higher SMARCE1 and PTK2 expression is associated with poor prognosis of breast cancer patients. **a** Kaplan-Meier analysis of recurrence-free survival (*RFS*) of breast cancer patients based on SMARCE1 and PTK2 expression using a dataset of 3554 breast tumor samples (http://kmplot.com/analysis/). To construct Kaplan-Meier curves all percentiles between the lower and upper quartiles were computed and the best performing threshold was used as cutoff to divide the patient cohort. The log-rank test *p* value was calculated by using RFS up to 15 years as end point. **b** Correlated expression of SMARCE1 and PTK2 in basal-like and luminal B subtypes of breast cancer according to the TCGA database. Tumors with PTK2 amplification were excluded from the correlation analysis. **c** Frequency of genetic alterations of SWI/SNF complex subunits in breast tumors according to the TCGA database

Having established a role of SMARCE1 in regulating metastatic potential of breast cancer cells through the HIF1A/PTK2 pathway, we sought to examine the clinical relevance of this finding by examining the relationship between the SMARCE1 and PTK2 expression and the clinical outcomes of breast cancer patients. By analyzing the expression data of 3554 breast tumor samples collected by an online Kaplan-Meier survival analysis tool [27], we found that higher expression of SMARCE1 or PTK2 is associated with shorter interval of relapse-free survival (RFS) of breast cancer patients (Fig. 11a). Next, we examined the relationship between the expression of SMARCE1 and PTK2 in breast tumors in The Cancer Genome Atlas (TCGA) database [28]. No significant correlation between SMARCE1 and PTK2 expression was detected when data from all breast tumors (*n* = 825) were included in the analysis. However, after tumors were stratified according to the intrinsic PAM50 subtype markers, a positive correlation between SMARCE1 and PTK2 expression was detected in the luminal B and basal-like cohorts (Fig. 11b), but not in the ERBB2-enriched or luminal A cohorts. Together, these observations suggest that higher expression of SMARCE1 and PTK2 increases risk of tumor relapse, and SMARCE1 likely plays a key role in regulating PTK2 expression in luminal B and basal-like tumors.

### Genomic alterations contribute to deregulation of SWI/SNF complexes in breast cancer

Despite the prevalent notion that SWI/SNF complexes are tumor suppressors [29], emerging evidence suggests that genetic alterations of subunit genes can produce aberrant SWI/SNF complexes with oncogenic function [30]. These findings promoted us to examine the nature and frequency of genetic alterations in SMARCE1 and other SWI/SNF subunits specifically in breast cancer by using the TCGA database. The result revealed that approximately 30 % of breast tumors harbor genetic alterations in one or more of the genes encoding SWI/SNF subunits (Fig. 11c). Intriguingly, gene amplification is the most frequent genetic event for multiple SWI/SNF subunits in breast cancer, rather than inactive mutations that were reported in other tumors [6]. SMARCE1 is the second most frequently amplified gene among all the SWI/SNF subunits. This finding implicates an oncogenic role of SWI/SNF complexes in human breast cancer. If a SWI/SNF subunit plays a critical role in promoting progression of breast cancer, we speculate a positive correlation between its expression and poor prognosis of breast cancer patients. Therefore, we examined the relationship between the expression of each SWI/SNF subunit and interval of RFS of breast cancer patients by using the online Kaplan-Meier survival analysis tool [27]. This analysis revealed that higher expression of nine (out of 31) SWI/SNF subunits (i.e*.*, BCL7C, BRD7, ACTL6A, SMARCE1, SMARCA5, SMARCAL1, SMARCA4, SS18, and SMARCC1) is associated with shorter interval of RFS of breast patients (log-rank *p* < 0.05, *n* = 3550). Overall these results from genetic and expression data analysis suggest that deregulation of SWI/SNF activity likely play a key role in promoting breast cancer progression.

## Discussion

Loss of adhesion to proper extracellular matrix (ECM) triggers a specific form of apoptosis termed anoikis in normal epithelial cells [31], which plays a fundamental role in morphogenesis of the mammary gland ductal system [32]. Circumvention of anoikis contributes to breast tumor development and is a prerequisite for metastases by allowing cells to survive loss or alterations of ECM [3, 18]. In mammary epithelial cells, the dynamic and magnitude of activation of pro-apoptotic genes (e.g*.*, BIM and BMF) or pro-survival proteins (e.g*.*, PTK2) were shown to play a critical role in determining the fates of detached cells, anoikis or survival [3, 18, 33, 34]. PTK2 is one of the most important signaling proteins recruited into focal adhesions upon cell-ECM contact, where PTK2 is rapidly autophosphorylated to enable the recruitment of other scaffold and signaling molecules to activate the downstream cell survival signaling pathways (e.g*.*, PI3K/Akt and MAPK/ERK pathways) [20, 34, 35]. Emerging evidence suggests that PTK2 plays a key role in protecting tumor cells against anoikis by sustaining survival signaling [18, 20]. However, how PTK2 is activated in detached cells have not been definitively resolved. Our studies revealed a key role of SMARCE1 in promoting survival of detached cells by facilitating rapid PTK2 transcription activation through HIF1A, the master regulator of hypoxia-responsive genes. Multiple kinases were reported to activate PTK2 through phosphorylation at Tyr576/577 in detached cells, including SRC family kinases and PTK6 [18, 36]. We found that SRC inhibition by dasatinib had no effect on viability of LM and BT549 cells under suspension. This observation suggests that PTK2 phosphorylation in detached breast cancer cells is likely sustained by a SRC-independent mechanism.

HIF1A activation under normal oxygen tension has been linked to anoikis resistance of breast cancer cells driven by ERBB2 oncogene [23], and circulating breast tumor cells from patients with metastatic breast cancer [21]. SWI/SNF complexes were shown to regulate transcription activation of a subset of HIF1A target genes under hypoxia [25]. However, it is unclear whether SWI/SNF complex is required for HIF1A transcription activity in detached cells under normal oxygen tension. Our study provides evidence suggesting an essential role of SMARCE1-based chromatin remodeling activity in breast cancer metastasis by facilitating HIF1A-mediated *PTK2* transcription activation in detached cells under normoxia. Future study is warranted to evaluate the anti-metastasis potential of approaches targeting SMARCE1-HIF1A interaction.

According to the database of Project Achilles that performed genome-wide pooled shRNA screens across hundreds of cancer cell lines to identify genes essential for tumor cell proliferation, SMARCE1 knockdown showed limited effect on proliferation of breast cancer cells [37]. Consistently, we found that SMARCE1 knockdown had no significant effect on proliferation of two breast cancer cell lines (i.e*.*, MDA-MB-231 and HCC38) in vitro and growth of orthotopic xenografts derived from MDA-MB-231 cells, while significantly enhanced anoikis sensitivity of breast cancer cells. These findings suggest that SMARCE1 is dispensable for proliferation of breast cancer cells under normal growth condition, but required for cells to survive anoikis. Whether SMARCE1 plays a role to protect cells against stress-induced cell death in general warrants further investigation.

In contrast to colorectal, melanoma and lung cancer where inactive mutations of SWI/SNF subunits frequently occur, breast tumors have low frequency of inactive mutations [6, 10, 11]. By inspecting the TCGA database we found that approximately 30 % breast tumors harbor genetic alterations (i.e*.,* gene amplification, deletion, and mutation) in one or more of the SWI/SNF subunits, with gene amplification being the most frequent event. This finding implicates an oncogenic role of SWI/SNF complexes in breast cancer. Consistently, we found that higher expression of several SWI/SNF subunits is associated with shorter interval of RFS of breast cancer patients. Our findings are likely relevant to basal-like and luminal B breast tumors, because a positively correlated expression between SMARCE1 and PTK2 is evident in these subtypes of tumors.

## Conclusion

This study establishes a novel role of SMARCE1 in regulating metastatic potential of breast cancer cells by promoting survival of detached cells through the HIF1A/PTK2 pathway.

## Abbreviations

BAF57, BRG/BRM-associated factor 57; BIM, BCL2-interacting mediator of cell death; BRG1, Brahma-related gene 1; BRM, Brahma gene; ChIP, chromatin immunoprecipitation; dsDNase, double-strand-specific DNase; ECM, extracellular matrix; EV, empty vector; FAK, focal adhesion kinase; KD, knockdown; LM, lung metastatic cell line derived from MDA-MB-231; MEGM, mammary epithelial cell growth medium; NSG, NOD.Cg-Prkdcscid Il2rgtm1Wjl/SzJ; NUSA, nucleosome-scanning assay; qPCR, quantitative polymerase chain reaction; RFS, recurrence-free survival; shRNA, short hairpin RNA; siRNA, short inhibitory RNA; SWI/SNF, switching defective/sucrose nonfermenting complex; TCGA, The Cancer Genome Atlas
